# Bone marrow mesenchymal stromal cells attenuate silica-induced pulmonary fibrosis potentially by attenuating Wnt/β-catenin signaling in rats

**DOI:** 10.1186/s13287-018-1045-4

**Published:** 2018-11-14

**Authors:** Enguo Zhang, Ye Yang, Shangya Chen, Cheng Peng, Martin F. Lavin, Abrey J. Yeo, Chao Li, Xiaoshan Liu, Yingjun Guan, Xinjing Du, Zhongjun Du, Hua Shao

**Affiliations:** 1 Department of Toxicology, Shandong Academy of Occupational Health and Occupational Medicine, Shandong Academy of Medical Sciences, No 18877 Jingshi Road, Lixia District Jinan, Jinan, 250062 Shandong People’s Republic of China; 2 School of Medicine and Life Sciences, University of Jinan-Shandong Academy of Medical Sciences, Jinan, Shandong People’s Republic of China; 3 Queensland Alliance for Environmental Health Sciences (QAEHS), The University of Queensland, Brisbane, Queensland Australia; 4 University of Queensland Centre for Clinical Research (UQCCR), The University of Queensland, Brisbane, Queensland Australia; 5 Department of Radiology, Shandong Tumor Hospital, Shandong Academy of Medical Sciences, Jinan, Shandong People’s Republic of China

**Keywords:** Silicosis, Bone marrow mesenchymal stem/stromal cells (BMSCs), Animal model, Pulmonary fibrosis, Transplantation, Cell therapy

## Abstract

**Background:**

Pulmonary fibrosis induced by silica dust is an irreversible, chronic, and fibroproliferative lung disease with no effective treatment at present. Previous studies have shown that early intervention with bone marrow mesenchymal stem/stromal cells (BMSCs) has positive effect on anti-pulmonary fibrosis caused by silica dust. However, early intervention using BMSCs is not practical, and the therapeutic effects of BMSCs advanced intervention on pulmonary fibrosis have rarely been reported. In this study, we investigated the effects of advanced transplantation (on the 28th day after exposure to silica suspension) of BMSCs on an established rat model of pulmonary fibrosis.

**Methods:**

Sprague Dawley (SD) rats were randomly divided into four groups including (1) control group (*n* = 6) which were normally fed, (2) silica model group (*n* = 6) which were exposed to silica suspension (1 mL of 50 mg/mL/rat), (3) BMSC transplantation group (*n* = 6) which received 1 mL BMSC suspension (2 × 10^6^ cells/mL) by tail vein injection on the 28th day after exposure to silica suspension, and (4) BMSC-CM (conditioned medium) transplantation group (*n* = 6) which received CM from the same cell number by tail vein injection on the 28th day after exposure to silica suspension. On the 56th day after exposure to silica suspension, we used computed tomography (CT), hematoxylin and eosin (H&E), and Masson’s trichrome staining to evaluate the changes in lung tissue. We examined the expression of epithelial-mesenchymal transition (EMT) and Wnt/β-catenin pathway-related proteins in lung tissue using immunohistochemistry and western blotting.

**Results:**

Successful construction of a pulmonary fibrosis model was confirmed by H&E and Masson’s trichrome staining on the 28th day after exposure to silica suspension. On the 56th day after exposure, pulmonary CT examination showed a relieving effect of BMSCs on silica-induced pulmonary fibrosis which was confirmed by H&E and Masson’s trichrome staining. Treatment of BMSCs increased the expression of epithelial marker proteins including E-cadherin (E-cad) and cytokeratin19 (CK19) and reduced the expression of fibrosis marker proteins including Vimentin (Vim) and α-Smooth actin (α-SMA) after exposure to silica suspension. Furthermore, we found that Wnt/β-catenin signaling pathway is abnormally activated in silica-induced pulmonary fibrosis, and exogenous transplantation of BMSCs may attenuate their expression.

**Conclusions:**

BMSC transplantation inhibits the EMT to alleviate silica-induced pulmonary fibrosis in rats and the anti-fibrotic effect potentially by attenuating Wnt/β-catenin signaling.

**ᅟ:**

ᅟ
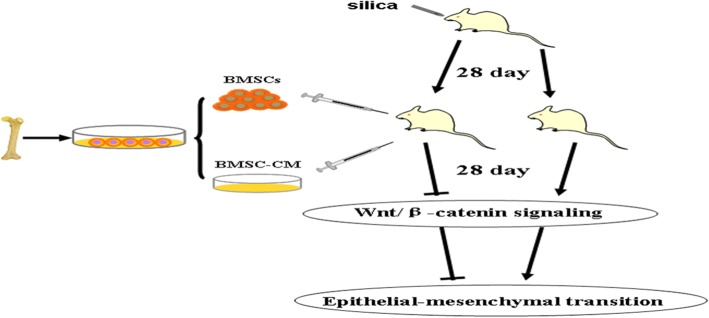

**Electronic supplementary material:**

The online version of this article (10.1186/s13287-018-1045-4) contains supplementary material, which is available to authorized users.

## Background

Silicosis is caused by long-term inhalation of dust containing excessive free silica during the production process and is specifically characterized by silicon nodule formation and diffuse pulmonary fibrosis [1]. There are currently no effective treatments to reverse or cure the pathological processes leading to a dismal prognosis of this disease [2, 3]. The transplantation of bone marrow mesenchymal stem/stromal cells (BMSCs) in animal models of fibrosis has shown repairing and protective effects [4–6]. As a burgeoning treatment, the BMSC therapy mainly includes differentiation [7] and paracrine mechanisms. However, it is becoming apparent that the protective effects related with mesenchymal stem/stromal cell (MSC) therapy are based less on in situ differentiation and more on paracrine or trophic factors [8, 9]. A previous study has reported the potential beneficial effect of BMSCs on silica-induced pulmonary fibrosis via paracrine mechanisms [10]. Paracrine secretion is a process during which vesicles, exosomes, and various cytokines are released by MSCs into the extracellular environment or in the medium (conditioned medium, CM) when they are cultured in vitro [11]. In support of this, a more recent study [12] found that BMSCs can improve pulmonary fibrosis induced by silica in vivo and in vitro, but more likely through a series of paracrine factors (HGF, KGF, BMP-7), rather than by a differentiation mechanism. These studies strengthen the hypothesis that BMSC or CM may be suitable for the treatment of pulmonary fibrosis. In many cases, when silicosis is diagnosed, the lungs may already have formed an advanced fibrosis due to substantial latency period of the disease. At present, most of the studies have focused on the anti-inflammatory and fibrous effect of early transplantation of BMSCs or CM before silicosis occurs. However, these findings are not really that relevant for clinical treatment, as patients present much later in the disease process than that modeled in these studies.

The pathogenesis of silicosis mainly involves a dynamic process including severe damage to alveolar epithelial cells, occurrence of epithelial-mesenchymal transition (EMT) and abnormal activation of myofibroblasts, resultant extracellular matrix (ECM) over-deposition, and consequent pulmonary fibrosis. Notably, EMT has been implicated in the pathogenesis of pulmonary fibrosis in response to epithelial injury. Lung epithelial cells undergoing EMT contribute to the lung fibroblasts in bleomycin (BLM)-induced lung fibrosis [13]. For idiopathic pulmonary fibrosis (IPF), it was proposed that a dysregulated communication between epithelial pulmonary components and mesenchymal after tissue injury is key to the irreversible process of fibrosis and tissue remodeling [14, 15]. In addition, BMSC treatment has been identified to reduce the injury of alveolar epithelial cells in vivo and attenuate silica-induced pulmonary fibrosis [12]. Previous studies have also reported that CM from BMSCs can inhibit the EMT process [16]. Notably, there are many signaling pathways involved in the regulation of EMT [17]. Previous studies indicated that the Wnt/β-catenin signaling pathway plays an important role in the EMT induction and the development of pulmonary fibrosis. The Wnt/β-catenin signaling pathway is highly conserved in the evolutionary process and well known for its functions during embryonic development and in body homeostasis [18]. Aberrant regulation of Wnt/β-catenin activation or loss of signaling has been linked to many diseases [19–22]. Increasing lines of evidence demonstrate that a dysregulated activation of Wnt signaling, particularly the Wnt/β-catenin signaling, is involved in the pathogenesis of lung diseases, such as IPF [23–26]. A study showed that BMSCs can attenuate alveolar macrophage apoptosis partially by suppressing the Wnt/β-catenin pathway [27]. Another study reported that the use of XAV939 small molecule can inhibit Wnt/β-catenin signaling and promote the differentiation of BMSCs into an epithelium-like phenotype in the co-culture system [28].

Therefore, in order to strengthen the clinical relevance of BMSCs for the therapeutic potential of pulmonary fibrosis, in this work, we aimed to explore the effects of advanced transplantation of BMSCs and their CM (on the 28th day after exposure to silica suspension) on the formed pulmonary fibrosis models. In addition, we observed the changes of the EMT process and Wnt/β-catenin signaling pathway-related proteins in rat lung tissue after exposure of silica suspension and transplantation of BMSCs.

## Materials and methods

### Experimental animals and experimental design

Specific pathogen-free (SPF) grade healthy adult male Sprague Dawley (SD) rats were purchased from Beijing Vital River Laboratory Animal Technology Co. Ltd. (the number for certificate of animals is SCXK (Jing) 2016-0011). All animals were housed in standard animal house conditions (temperature 18~24 °C; humidity 45%; light and dark alternating time 12 h:12 h). Animals were kept on conventional conditions. Food and water were provided ad libitum. All animals in this study were treated according to the protocols evaluated and approved by the experimental animal ethical committee of Shandong Academy of Medical Sciences.

**Fig. 1:**
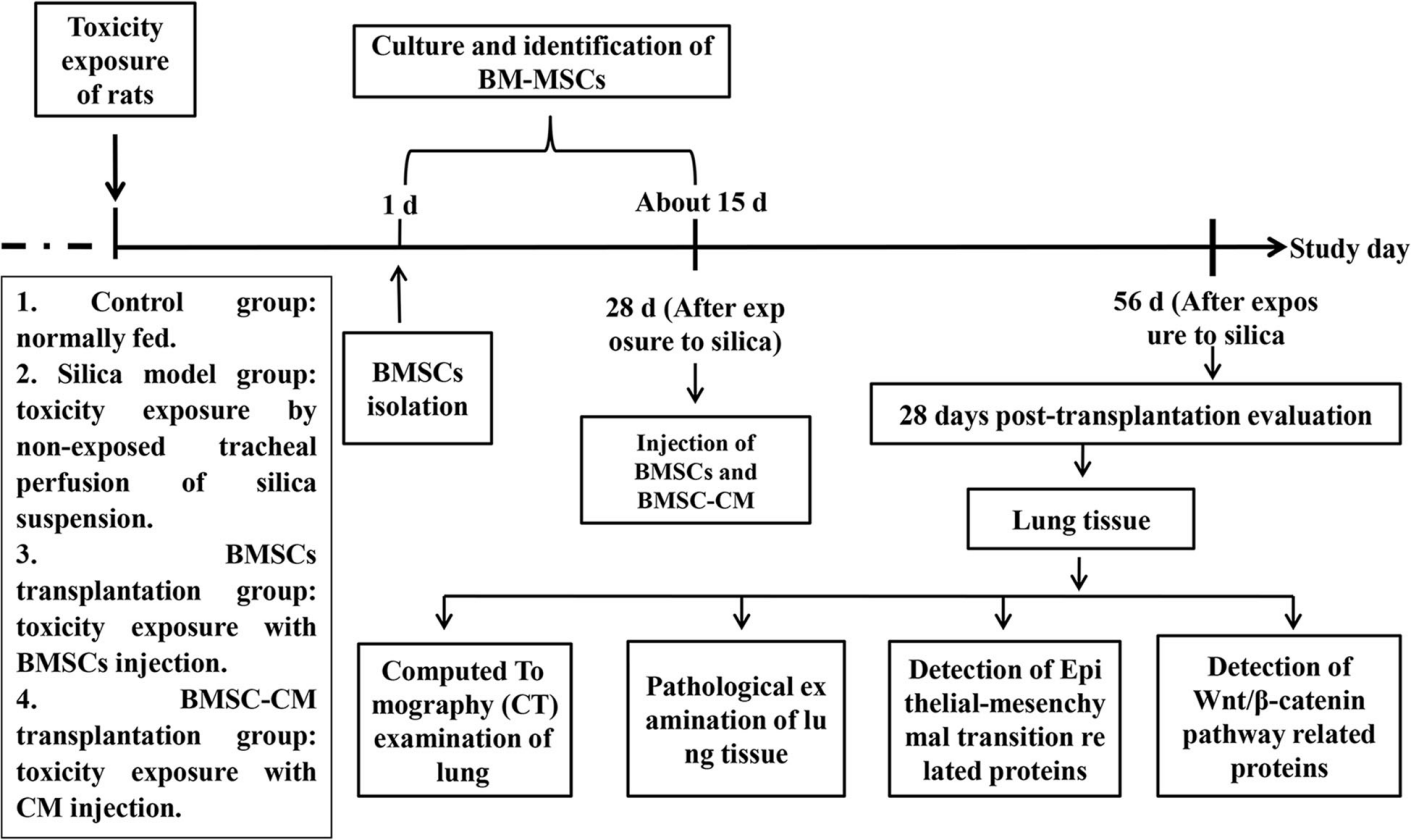
Experiment design to study the anti-fibrosis effects of advanced transplantation of BMSCs or BMSC-CM in adult rats

Twenty four rats (6~8 weeks old) were randomly divided into four groups including (1) control group (*n* = 6) which were normally fed, (2) silica model group (*n* = 6) which were exposed to silica suspension (1 mL of 50 mg/mL/rat), (3) BMSC transplantation group (*n* = 6) which received 1 mL BMSC suspension (2 × 10^6^ cells/mL) by tail vein injection on the 28th day after exposure to silica suspension, and (4) BMSC-CM transplantation group (*n* = 6) which received CM from the same cell number by tail vein injection on the 28th day after exposure to silica suspension. All animals were examined for pulmonary CT on the 56th day after exposure to silica suspension. Then, the rats were sacrificed by intraperitoneal injection of 3% pentobarbital sodium anesthetic overdose and lung tissue was harvested for subsequent experiments. The technical diagram of this experiment is shown in Fig. 1.

### Isolation, culture, and identification of BMSCs

BMSCs were isolated and cultured by whole bone marrow adherence method as previously described [29]. Briefly, the 4-week-old male adult SD rats (*n* = 3) were sacrificed by 3% pentobarbital sodium anesthetic overdose. After being put in 75% ethanol for about 5~10 min, the bilateral femur and tibia were isolated in the super-clean bench and washed three times with PBS containing 10% 100 IU/mL penicillin/streptomycin (Biological Industries, USA). After transferring into a dry and clean Petri dish, the metaphyseal was cut and the bone marrow cavity was exposed. Fresh BMSC suspensions were collected by flushing the medullary cavity of rat femurs with Dulbecco’s modified Eagle’s medium (DMEM; ThermoFisher Scientific, USA), and this step was repeated three times until the bone marrow cavity turns white. DMEM complete medium was supplemented with 10% fetal calf serum (Biological Industries, USA) and 1% 100 IU/ml penicillin/streptomycin (Biological Industries, USA). The bone marrow solution was subsequently filtered once using a 70-μm filter, and purified cells were dispersed in cell culture flasks (ThermoFisher Scientific, USA), grown in DMEM complete medium and then cultured at 37 °C in a CO_2_ incubator (Labserv CO-150; ThermoFisher Scientific, USA). Following culture for 48 h, fresh media was added and non-adherent cells were removed. Medium was replaced every day, and the growth status of the cells was monitored using an inverted microscope (IX70; Olympus, Japan). Adhered cells were allowed to grow to 80~90% confluency and then trypsinized for subculture. The bone marrow cells at the third generation were harvested and ready for characterization.

BMSCs were identified by flow cytometry (FCM) with CD90, CD73, CD44, CD45, and CD11b antibodies. All antibodies were purchased from BD (Becton Dickinson and Co., USA) and used in accordance with the instructions of the manufacturer. Non-specific staining was controlled by the use of isotype-matched antibodies. BMSCs at the third generation were harvested by trypsin-EDTA (ThermoFisher Scientific, USA). Cells collected after digestion were centrifuged by 1000 rpm and washed with PBS. BMSC suspensions were incubated with antibodies and their isotype controls (1:100) at 4 °C for 30 min. After incubation, flow cytometry was carried out and cells were analyzed using Flow Jo software (Flow Jo, LLC, USA).

BMSCs were differentiated into osteogenic, adipogenic, and chondrogenic lineages. After subculturing to third generation, when the cells had grown to about 80%, the culture medium was replaced with osteogenic, adipogenic, and chondrogenic differentiation complete medium (using osteogenic, adipogenic, and chondrogenic differentiation kits; Thermo Fisher Scientific, USA). After induction of the mineralized osteogenic cultures for 21 days, accumulation of calcium, intracellular lipids, and mucopolysaccharide were detected by staining with alizarin red S, oil red O, and toluidine blue (Sigma-Aldrich, USA), respectively.

### Collection and concentration of CM

When the third generation of BMSCs achieved 80–90% confluence, cells were starved with serum-free medium for 24 h. The supernatant of BMSCs was collected, centrifuged at 300×*g* for 5 min and filtered using a 0.22-μm filter (Millipore, USA). Then, the supernatant was transferred to a Millipore Amicon Ultra-15 ultrafiltration centrifuge tube (100 kDa cutoff; Millipore, USA), centrifuged at 4 °C, by 5,000×*g* for 40 min, and the precipitate was used as the BMSC-CM. This was stored in a refrigerator at − 80 °C for subsequent experiments.

### Silica-induced silicosis in rats

The rat model of silicosis was induced with 1 mL of one-off infusing silica suspension using the non-exposed intratracheal instillation. The silica (approx. 80% between 1 and 5 μm in diameter, Sigma-Aldrich, USA) was autoclaved and made a suspension at 50 mg/mL in sterile normal saline. Rats were anesthetized with 3% pentobarbital sodium and were fixed on the operating table after anesthesia. Rat necks were irradiated with a cold light source, and the glottis was identified as the bright spot opening and closing with breathing. When the glottis was open, the indwelling venous needle was inserted into the trachea. Subsequently, silica suspension was injected rapidly into the trachea. Finally, the rat was gently shaken for about 1 min to allow for uniform distribution of the suspension in the lungs. The rats in the control group were perfused with 1.0 mL of sterile normal saline.

### BMSC and BMSC-CM administration

BMSCs were re-suspended at a concentration of 2 × 10^6^ cells/mL for transplantation. Briefly, on the 28th day after intratracheal administration of silica suspension, rats were anesthetized with 3% pentobarbital sodium. The rat tail was sterilized by 75% ethanol. To the BMSC transplantation group, 1 ml BMSC suspension (2 × 10^6^ cells/mL/rat) was injected within 2 min through a tail vein puncture. To BMSC-CM transplantation group, CM secreted from 2 × 10^6^ cells was injected within 2 min through a tail vein puncture.

### CT examination of the lung (on the 56th day after exposure to silica suspension)

Rats were anesthetized with 3% pentobarbital sodium and placed in the supine position to fix the rat on the plate to extend the extremities and fully expose the scanning area of the lungs. Sixty-four slice spiral CT (GE Discovery CT750 HD, USA) was used to scan the range of the neck to the diaphragm of the rats.

### Histopathological examination

Rats were sacrificed by intraperitoneal injection of 3% pentobarbital sodium anesthetic overdose. The lungs of rats were isolated quickly, and the left lung was fixed in 4% (*v*/*v*) paraformaldehyde. After embedded in paraffin following the standard histology procedure, tissues were serially sectioned at a thickness of 5 μm and then mounted onto glass slides. Tissues on the glass slides were stained with hematoxylin and eosin (H&E) for histopathological evaluation and stained with Masson’s trichrome stain for fibrotic examination. The whole section was then scanned, and a semi-quantitative score of pulmonary fibrosis was performed according to the modified Ashcroft scale [30].

### Hydroxyproline assay

Hydroxyproline content was used as an index of fibrosis in the lung tissue. To assess the degree of collagen deposition, the hydroxyproline contents in the lung tissues were measured by using a hydroxyproline kit from Nanjing Jian Cheng Institute (Nanjing, China) following instructions from the manufacturer. All procedures were done according to the manufacturer’s instruction, and the absorbance was determined at 550 nm. The results were calculated as micrograms of hydroxyproline per milligram of wet lung weight.

### Western blot assay

Lung tissue of rats was washed with cold PBS two to three times, cut into small pieces, and lysed with RIPA buffer. After being re-suspended in homogenization buffer, the tissues were centrifuged for 10 min (12,000 rpm, 4 °C). Supernatants were collected as protein suspension. The samples were subjected to sodium dodecyl sulfate polyacrylamide gel electrophoresis (SDS-PAGE). Then, protein samples were transferred to PVDF membranes (200 mA, 1 h). The membranes were blocked with 5% fat-free dry milk (prepared with 0.5% TBSP) for 1 h at room temperature. Then, blots were incubated with the following primary antibodies overnight at 4 °C: anti-GSK3β (CST, USA), anti-P-GSK3β (CST, USA), anti-β-catenin (CST, USA), anti-Cyclin D1 (Abcam, USA), anti-E-cadherin (E-cad; Santa Cruz, USA), anti-cytokeratin19 (CK19; Santa Cruz, USA), anti-Vimentin (Vim; Santa Cruz, USA), anti-α-Smooth muscle actin (α-SMA; CST, USA), and anti-β-actin (Santa Cruz, USA). Following this, they were washed three times with TBST at room temperature on a decolorization shaker for 5 min each time. The membranes were incubated with secondary antibodies (Santa Cruz, USA) at 1:3000 dilutions for 0.5 h at room temperature. After being washed by TBST, the blots were treated with enhanced chemiluminescence (ECL detection kit, Pierce). The optical density of the blots was analyzed with AlphaEaseFC software (Alpha Innotech, USA). Experiments were performed in triplicate.

### Immunohistochemical analysis

Rat lungs were placed in 4% (*w*/*v*) paraformaldehyde and processed for paraffin embedding. The paraffin sections were dewaxed and rehydrated, followed by antigen retrieval. Endogenous peroxidase was inactivated with 3% H_2_O_2_ for 25 min at room temperature. Sections were incubated in 3% BSA solution for 30 min to block non-specific binding. Next, sections were incubated overnight at 4 °C with rabbit anti-P-GSK-3β (Servicebio; 1:400) or anti-β-catenin (CST; 1:500) and incubated with the relevant second antibody (Servicebio; HRP marker) at room temperature for 50 min. DAB was used to reveal the immunohistochemical reaction, and hematoxylin was used to stain nuclei. Positive results were expressed as brownish yellow. We used PBS substituted for the primary antibody as a negative control. Pannoramic MIDI (3D HISTECH) tissue slice scanner was used to scan followed by image analysis with the Pannoramic viewer software.

### Statistical analysis

Data were expressed as mean ± SD, and statistical analysis was performed using one-way analysis of variance (ANOVA). Multiple comparisons were performed using the Bonferroni method and using the Dunnett T3 test when the variance was irregular. All the statistical analyses used SPSS software (IBM SPSS Statistics 19.0, USA). All experiments were repeated three times and results were considered statistically significant at *p* values < 0.05.

## Results

### Growth state and identification of BMSCs

**Fig. 2:**
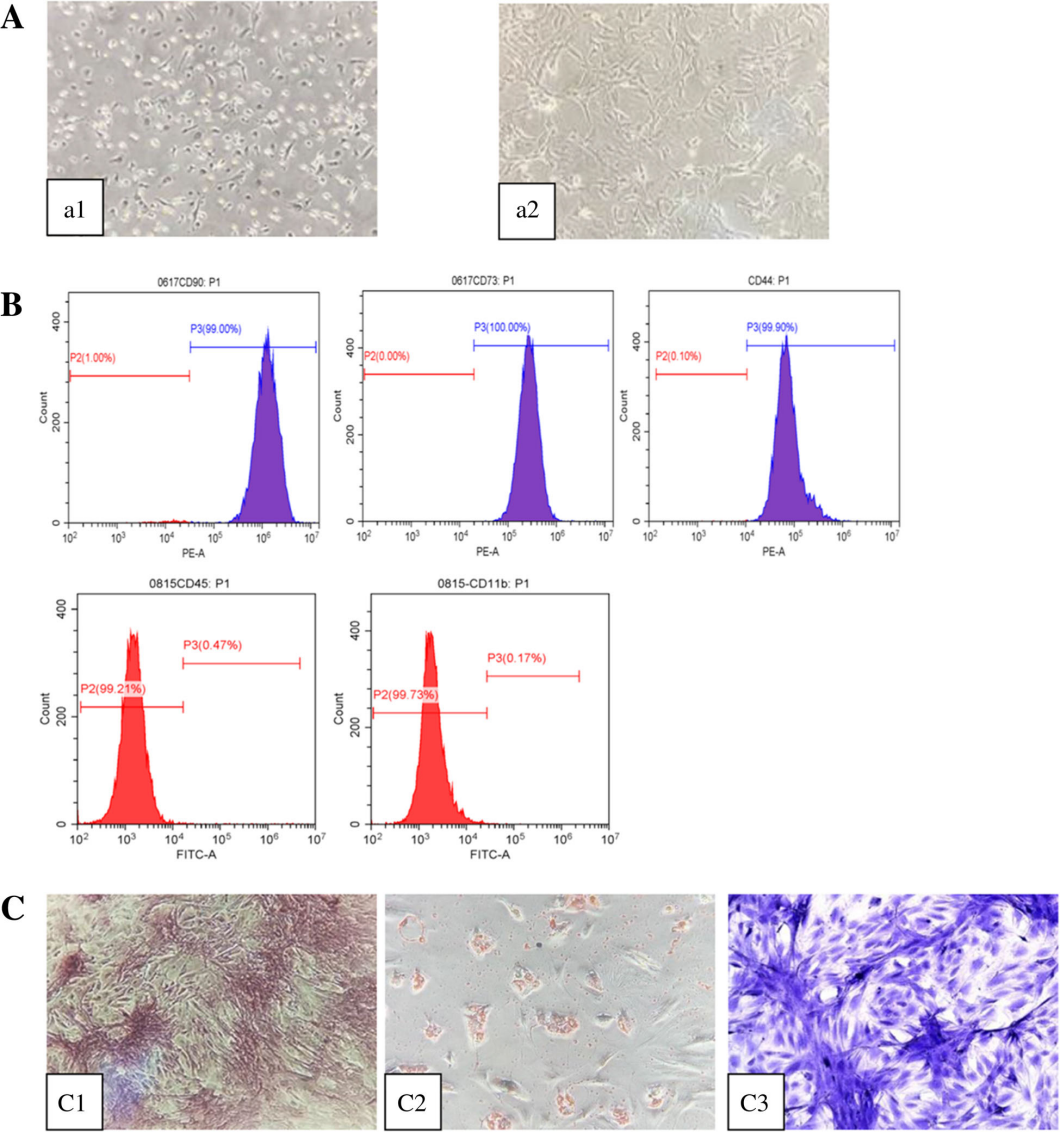
Cultivation and characterization of rat BMSCs. **a** Morphology of bone marrow cell in passage 1 (a_1_, × 100) and passage 3 (a_2_, × 100). **b** Surface markers of BMSCs. The immunophenotype of rat BMSCs was analyzed by flow cytometry. Most cells expressed CD90, CD73, and CD44, but were CD45-negative and CD11b-negative. **c** Multipotency of BMSCs. Rat BMSCs could differentiate into osteogenic (c_1_, × 200), adipogenic (c_2_, × 200), and chondrogenic (c_3_, × 200) lineages when cultured in differentiation medium

Primary BMSCs were initially non-homogeneous in morphology, but by 2 to 3 days became rounded and grew well. These cells were reduced in numbers after a single passage. After removal of hematopoietic stem cells and other non-adherent cells were removed, the remaining cells were homogeneous, spindle shape, showing large flake radial or swirl-like colonies. Flow cytometric analysis demonstrated that third generation cells expressed of CD90, CD73, and CD44 (90.00%, 100.00%, and 99.90% respectively), with little evidence of the expression of CD45 and CD11b (0.47% and 0.17% respectively). Meanwhile, osteogenic, adipogenic, and chondrogenic differentiation of cells at the third generation all succeeded. Thus, these results confirmed that the vast majority of cells in the third generation were BMSCs (Fig. 2).

### The general morphology of lung tissue

The general state of lung tissue in each of the groups was observed by the naked eye. The lung tissue in the control group was smooth and red in color, while in contrast, a significant swelling and congestion in the lung surface of the rats in the silica model group was observed, accompanied by large pathological changes of white granular pulmonary fibrosis, especially in the right upper lobe. Varying degrees of swelling and congestion, accompanied by mild changes of white granular pulmonary fibrosis were seen in the BMSCs and BMSC-CM transplantation groups (see Additional file 1).

### CT imaging examination of lung in each group

**Fig. 3:**
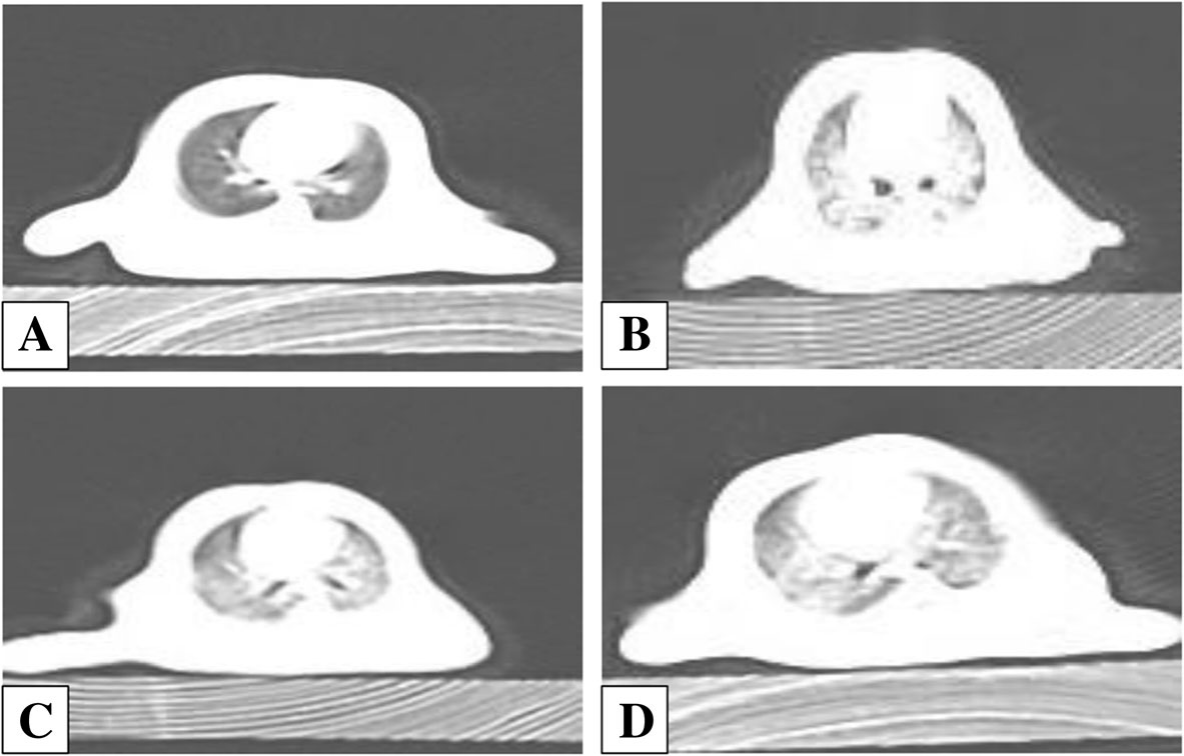
CT examination of the lung of the rats from each group: **a** control group, **b** silica model group, **c** BMSC transplantation group, and **d** BMSC-CM transplantation group

Lung fields of rats in the control group were clear and lucent with clear lung markings and no obvious high-density shadow. Different-sized granular high-density shadows or reticular fibrous shadows were found diffusely distributed in the lungs of the rats in silica model group. Rat lungs in BMSCs or BMSC-CM transplantation group showed scattered distribution granular high-density shadow and small pieces of reticular fibrous shadow, but the size of the particles and area of involvement were smaller than those in silica model group (Fig. 3).

### Pathological changes and hydroxyproline levels of lung tissue

**Fig. 4:**
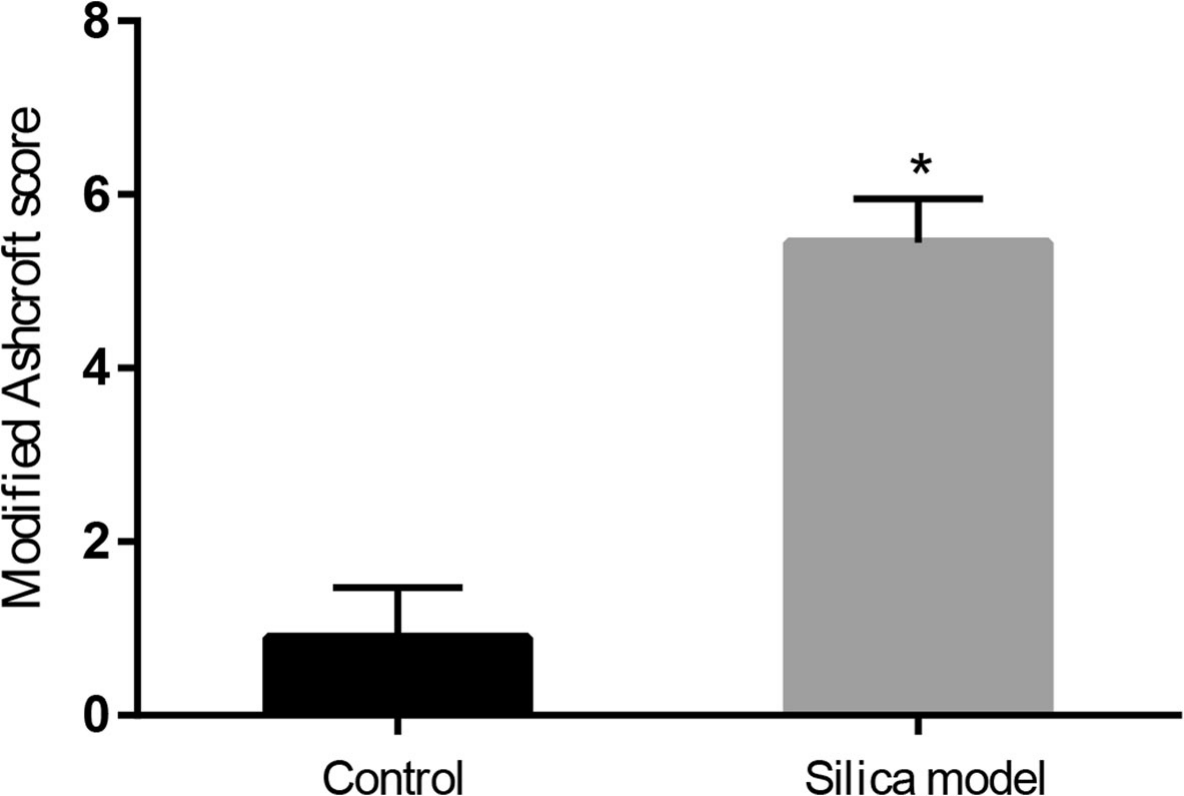
Severity of pulmonary fibrosis evaluated by modified Ashcroft score (on the 28th day after exposure to silica suspension). Data are presented as mean ± SD. ^*^p < 0.05 compared with the control group

To determine whether the silicosis pulmonary fibrosis model was constructed on the 28th day (BMSCs and CM transplantation time) after exposure to silica suspension, we performed H&E and Masson trichrome staining in the control group and silica model group (see Additional file 2). Red clusters in H&E staining indicated extensive infiltration of inflammatory cells, and blue areas in Masson’s trichrome staining indicated collagen deposition in the rat lungs. The results showed that the alveolar structure of control group rat was intact with no inflammatory cell infiltration and fibrosis. In contrast, the alveolar structure of the rats in the silica model group showed varying degrees of damage, accompanied by inflammatory cell aggregation and collagen fiber deposition. Semi-quantitative analysis of lung histopathology using the modified Ashcroft scale revealed that the degree of pulmonary fibrosis in the silica model group was significantly higher than that in the control group (Fig. 4; *p* < 0.05). These results showed that the pulmonary fibrosis model was successfully constructed on the 28th day after exposure to silica suspension.

**Fig. 5:**
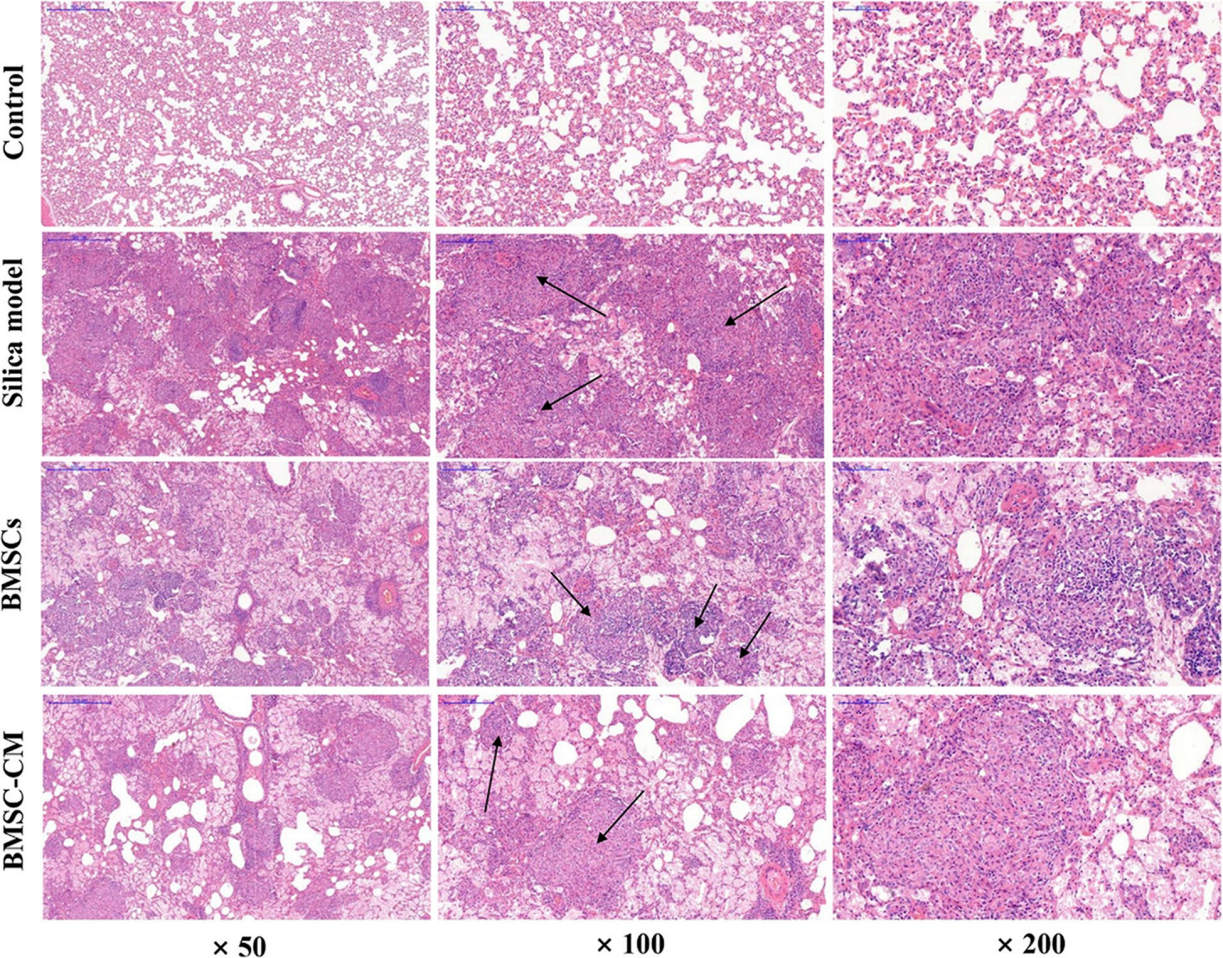
Lung histopathology on the 56th day after exposure to silica suspension in each group: H&E staining of lung tissue, magnification × 50, × 100, and × 200 (arrows indicate massive aggregation of inflammatory cells and fibrotic lesions)

**Fig. 6:**
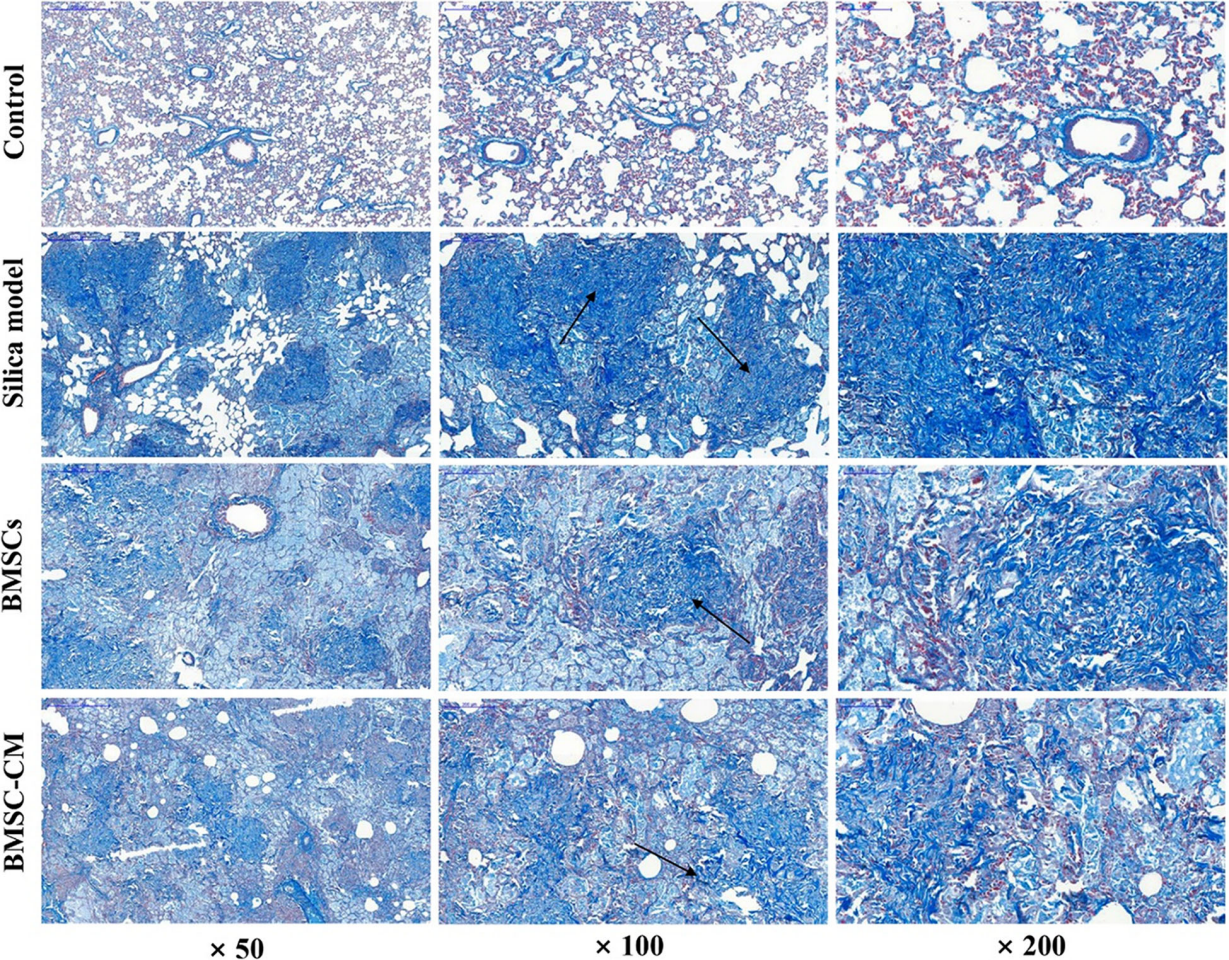
Lung histopathology on the 56th day after exposure to silica suspension in each group: Masson staining of lung tissue, magnification × 50, × 100, and × 200 (arrows indicate the deposition of collagen fibers)

**Fig. 7:**
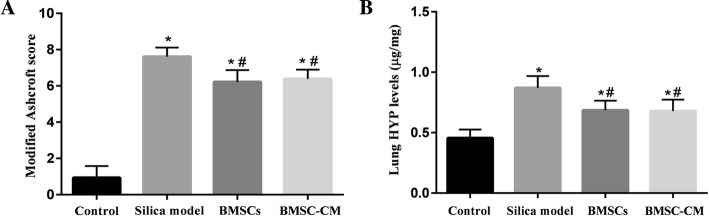
**a** Severity of pulmonary fibrosis evaluated by modified Ashcroft score (on the 56th day after exposure to silica suspension). **b** Hydroxyproline content in lung tissues of each group (on the 56th day after exposure to silica suspension). Data are presented as mean ± SD. *o < 0.05 compared with the control group, ^#^*p* < 0.05 compared with the silica model group

The rats were sacrificed on the 56th day (28 days after transplantation of BMSCs and CM), and the lung tissues of each group were histologically examined. The results of H&E and Masson trichrome staining were demonstrated in Figs. 5 and 6, respectively. Histological examination of lung tissue demonstrated that the alveolar structure of the control group was intact with no obvious inflammatory cell infiltration and fibrosis. However, in the lungs exposed to silica suspension, the alveolar structure in the silica model group was markedly destroyed, accompanied by a large area of inflammatory cell aggregation and silicon nodules (Fig. 5). Compared with the control group, dark blue-stained areas were obviously increased in the silica model group (Fig. 6). After BMSCs or BMSC-CM transplantation, inflammatory cell aggregation and collagen fiber deposition was evident, but the area of aggregation and deposition was decreased and weaker compared to the silica model group (Figs. 5 and 6). The result of modified Ashcroft score evaluation showed that the degree of pulmonary fibrosis in the silica model group was significantly higher than that in the control group (Fig. 7a; *p* < 0.05). Furthermore, pulmonary fibrosis was attenuated to a certain degree after transplantation compared to the silica model group (Fig. 7a; *p* < 0.05). We evaluated collagen deposition in the lung tissues by analyzing the hydroxyproline content. Hydroxyproline content (Fig. 7b) in the silica group was significantly higher than that in the control group (*p* < 0.05) at 56 days after silica instillation. However, the hydroxyproline level was decreased in the other two groups (*p* < 0.05).

### Abnormal EMT after exposure to silica suspension

**Fig. 8:**
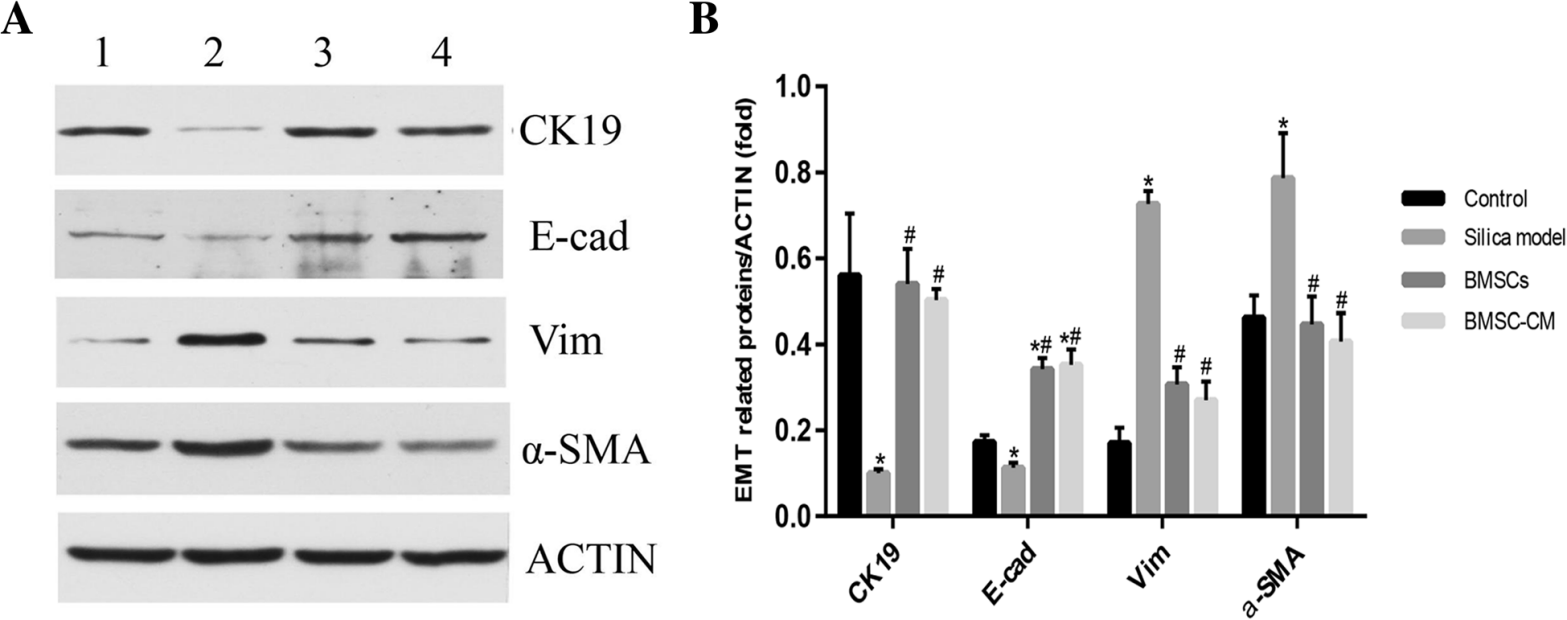
The expression levels of CK19, E-cad, Vim, α-SMA, and β-actin proteins of lung tissue in rats on the 56th day after exposure to silica suspension. **a** Western blot analysis of CK19, E-cad, Vim, α-SMA, and β-actin in lung tissue; (1) control group, (2) silica model group, (3) BMSC transplantation group, and (4) BMSC-CM transplantation group. **b** Relative ratio of CK19, E-cad, Vim, and α-SMA proteins of lung tissue. *p < 0.05 vs. control group; ^#^*p* < 0.05 vs. silica model group

To determine whether silica suspension induces abnormal activation of EMT, we measured the expression levels of a number of protein markers associated with this process. After instillation of silica suspension, the expression level of both CK19 and E-cad proteins were decreased compared with the untreated control (Fig. 8; *p* < 0.05). On the other hand, the expression level of Vim and α-SMA proteins were increased (*p* < 0.05). However, injecting BMSCs or CM via the tail vein on the 28th day after instillation of the silica suspension, the expression levels of CK19 and E-cad were increased compared with the silica model group (*p* < 0.05). Furthermore, the expression levels of Vim and α-SMA proteins were decreased in transplantation groups (*p* < 0.05).

### Wnt/β-catenin signaling pathway is abnormally activated in silica-induced pulmonary fibrosis

**Fig. 9:**
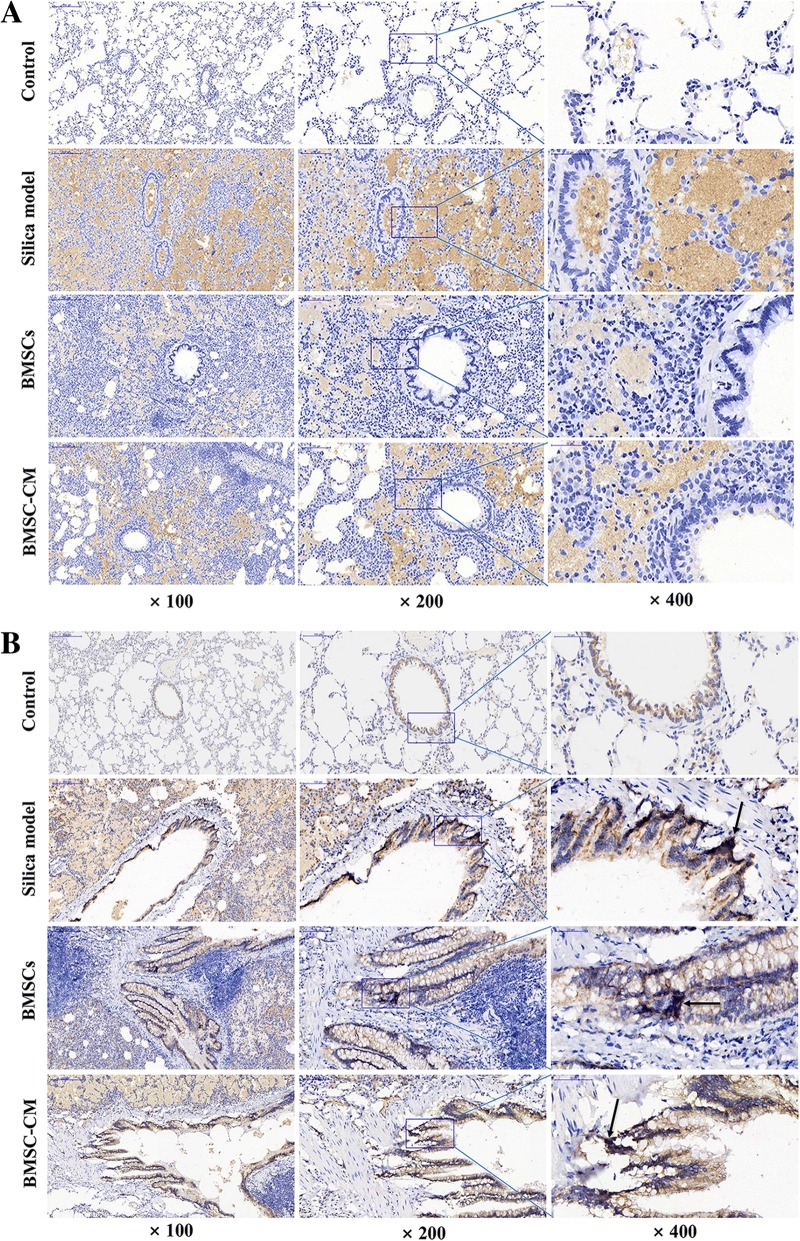
Immunohistochemical expression and localization of P-GSK-3β (**a**) and β-catenin (**b**) in lung tissues of each group, magnification × 100, × 200, and × 400. **a** P-GSK-3β is deposited in the cytoplasm of the silica model group and transplantation group. **b** β-catenin is deposited in the cytoplasm of the silica model group and transplantation group. Aggregation staining of β-catenin in nucleus was indicated by the arrow

**Fig. 10:**
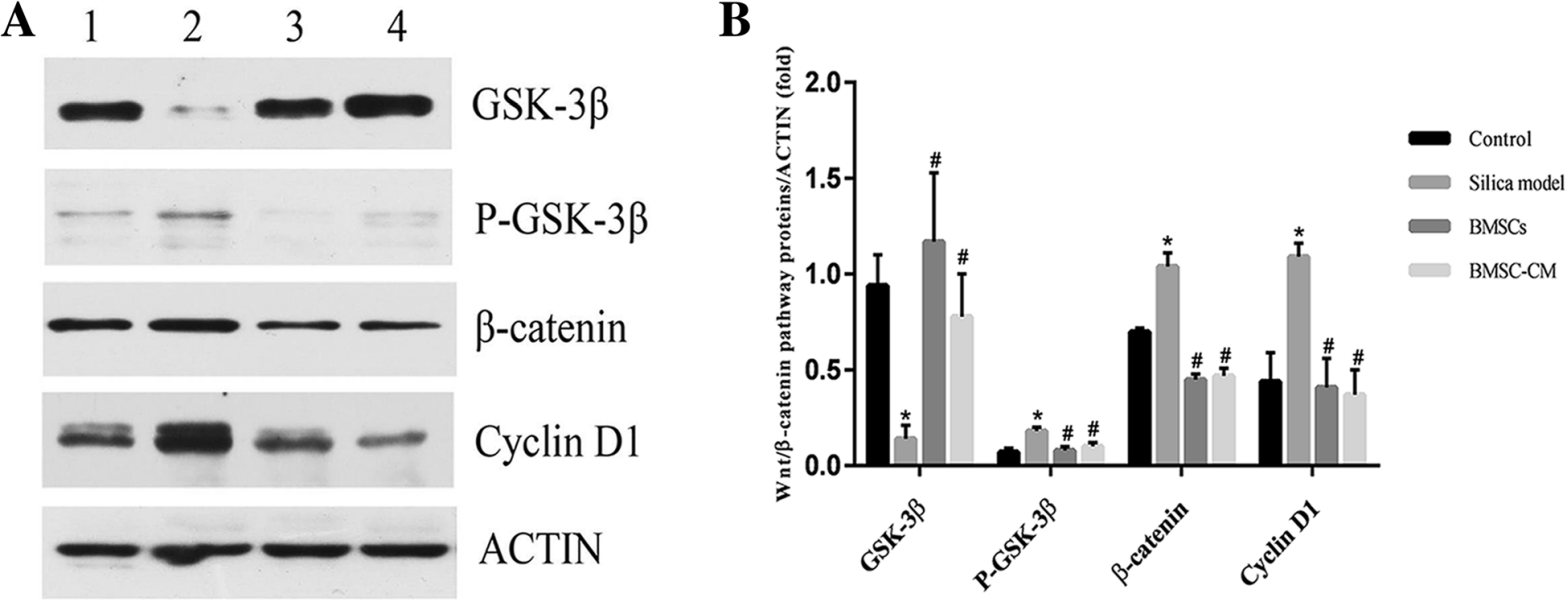
The expression levels of GSK-3β, P-GSK-3β, β-catenin, Cyclin D1, and β-actin proteins of lung tissue in rats on the 56th day after exposure to silica suspension. **a** Western blot analysis of GSK-3β, P-GSK-3β, β-catenin, Cyclin D1, and β-actin of lung tissue; (1) control group, (2) silica model group, (3) BMSC transplantation group, and (4) BMSC-CM transplantation group. **b** Relative ratio of GSK-3β, P-GSK-3β, β-catenin, and Cyclin D1 proteins of lung tissue. *p < 0.05 vs. control group; ^#^*p* <0.05 vs. silica model group

Previous studies have provided evidence that Wnt/β-catenin signaling is abnormally activated under a variety of conditions causing fibrosis [31–33]. In this study, we found that β-catenin was expressed in lung tissue in each group, but the level of expression in the cytoplasm after exposure to silica suspension was increased over that in controls (Fig. 9b). Furthermore, aggregation staining of β-catenin in nucleus was observed in the silica model group and BMSCs and BMSC-CM transplantation groups (Fig. 9b). For P-GSK3β protein, we observed almost no expression in the control group but in the silica model group, we found that P-GSK3β is abundantly deposited in the cytoplasm (Fig. 9a). However, P-GSK3β expression levels were reduced after transplantation of BMSCs or CM (Fig. 9a). In order to confirm the changes in expression of Wnt/β-catenin signaling pathway-related proteins, we used Western blot to detect the expression level of GSK-3β, P-GSK-3β, β-catenin, and Cyclin D1 protein in lung tissue (Fig. 10). The results demonstrate that the expression level of GSK-3β was decreased in the silica model group compared with the control group, while the expression levels of P-GSK-3β, β-catenin, and Cyclin D1 were increased (*p* < 0.05). However, after transplantation of BMSCs or CM on the 28th day, the expression level of GSK-3β was increased compared with the silica model group, while the expression levels of P-GSK-3β, β-catenin, and Cyclin D1 were decreased (*p* < 0.05).

## Discussions

Recently, much attention has been paid to stem cell therapy in experimental pulmonary fibrosis models [12, 34–36]. However, most of these studies explored the anti-fibrotic effects of early transplantation [37]. Therefore, we explored the effects of BMSCs and CM transplantation on advanced lung fibrosis and results showed that BMSCs and CM transplantation (on the 28th day after exposure to silica suspension) can relieve pulmonary fibrosis in rats. We found that transplantation can reduced pathological changes to the lungs of silica-treated animals, restored epithelial characteristics, and decreased mesenchymal features as revealed by expression of specific proteins. Furthermore, attenuation of the Wnt/β-catenin signaling pathway may be one of the mechanisms involved.

Firstly, we successfully developed the pulmonary model of fibrosis by exposing rats to silica suspension for 28 days (Fig. 4; Additional file 2), and then transplanted BMSCs or CM at this time. After transplantation for 28 days, rat lungs in BMSCs or BMSC-CM transplantation group showed scattered distribution granular high-density shadow and small pieces of reticular fibrous shadow, but the area of involvement were smaller than that in silica model group (Fig. 3). In addition, we found that after BMSCs or CM transplantation, inflammatory cell aggregation and collagen fiber deposition was evident, but the area of aggregation and deposition was decreased and weaker to a certain degree compared to silica model group (Figs. 5 and 6). Notably, previous studies have reported that the effect of BMSC transplantation (on the 28th day after exposure to silica suspension) for 14 days is not obvious [38], but after 56 days of transplantation, there is a certain therapeutic effect [39]. Different results from these studies may be due to several factors including the dose of silica dust, the number of transplanted cells, and the post-transplant treatment time.

When the lungs are exposed to silica particles for a long time, healthy epithelial cells are damaged and proliferation decreases; however, the ability of alveolar epithelial cells to repair themselves is limited. Inadequate epithelium repair is followed by an abnormal healing process including inflammatory cytokine production, fibroblast growth gradually predominant, and myofibroblast activation [10, 40–42]. EMT, a process by which differentiated alveolar epithelial cells undergo a phenotypic conversion that gives rise to the matrix-producing fibroblasts and myofibroblasts, is recognized as an important component of tissue fibrogenesis [17, 43]. EMT has changes not only in cell phenotype, but also in cell markers, mainly manifested as the loss of epithelial cell markers, including the downregulation of expression levels of proteins such as E-cad and CK19, and the obtainment of interstitial cell markers, including the upregulation of expression levels of proteins such as Vim and α-SMA [44–49]. Our results showed that the expression levels of the classical epithelial markers, E-cad and CK19, decreased in the silica model group compared with the control group, indicating the loss of epithelial cells whereas expression levels of Vim and α-SMA increased, supporting increased fibroblast activity. These abnormalities in protein expression point to increased EMT. On the other hand, exogenous transplantation of BMSCs or CM showed the opposite trend (Fig. 8), which suggests that exogenous transplantation can inhibit the development of EMT. The inhibition of EMT and protection of epithelial cells from damage may be an important cause of alleviating fibrotic progress by BMSCs.

The mechanisms involved in the formation of pulmonary fibrosis are complex, and a variety of signaling pathways may be involved and intricately linked at different levels in the process. Interestingly, EMT can be induced by some cytokines, whose expression can be tuned by complex regulatory loops with β-catenin signaling and which are potentially involved in the pathogenesis of pulmonary fibrosis [26, 50, 51]. Previous studies have shown that Wnt/β-catenin signaling is abnormally activated in a variety of causes of lung fibrosis, and the use of inhibitors to attenuate Wnt/β-catenin signaling can inhibit the progression of pulmonary fibrosis [52, 53]. As a switching signal molecule of the Wnt signaling pathway, nuclear transfer of β-catenin is a hallmark of Wnt/β-catenin signaling activation [26]. Previous study has reported that blockage of the Wnt/β-catenin signaling by the small interfering RNA for β-catenin was found to attenuate pulmonary fibrosis [54], suggesting that β-catenin may be an effective target for anti-fibrosis therapy. In this study, we found that β-catenin was expressed in lung tissue in each group, but the level of expression of this protein in the silica model group and transplantation group was increased over that in controls (Fig. 9b). Furthermore, nuclear transfer and aggregation staining of β-catenin was observed in the silica model group and transplantation groups (Fig. 9b), indicating that Wnt/β-catenin signaling may be activated after exposure to silica. In addition, we examined the expression of GSK-3β, P-GSK-3β, β-catenin, and Cyclin D1 protein by Western blot assay in order to further investigate the effect of the signaling attenuation after transplantation in rats with oral tracheal intubation with silica suspension. Among them, GSK3β is a negative regulator of Wnt/β-catenin pathway, and Cyclin D1 is a downstream target gene. We found that the expression level of GSK-3β was decreased in the silica model group compared with the control group, while the expression levels of P-GSK-3β, β-catenin, and Cyclin D1 were increased (Fig. 10). Therefore, the Wnt/β-catenin signaling pathway may be abnormally activated after exposure to silica suspension, which can inhibit the phosphorylation of glycogen synthase kinase-3β (GSK-3β)-mediated, resulting in a large amount of P-GSK3β deposition (Fig. 9a), thereby inhibiting the degradation of β-catenin in the cytoplasm and causing it to aggregate in large amounts (Fig. 9b). Then, a large amount of β-catenin accumulated in the cytoplasm can be transferred to the nucleus and interaction with T cytokine factor/lymphocyte enhancer factor − 1 (TCF/LEF-1) nuclear transcription coactivator regulates the downstream target genes and makes them overexpressed. Importantly, increased expression of GSK-3β after BMSCs or CM transplantation indicates that GSK-3β-mediated phosphorylation is enhanced (Fig. 10), promotes β-catenin degradation, and reduces its deposition in the cytoplasm (Fig. 9b), thereby reducing the expression level of downstream target gene (Fig. 10), which may be related with the development of EMT and subsequent fibrosis [7, 13, 14]. Notably, EMT can be induced by many stimuli and a variety of signaling pathways may be involved and intricately linked at different levels in the process of regulating this process, such as Wnt/β-catenin signaling, TGFβ/Smad, and integrin/ILK [17, 55, 56]. Although the precise molecular mechanism by which BMSCs are involved in silica-induced pulmonary fibrosis could not be defined in this study, our findings can contribute to decipher the mechanisms involved in the pathogenesis of pulmonary fibrosis and might also help in the search for new strategies to treat irreversible lung remodeling. Meanwhile, we realized that this is a preliminary work of the BMSCs which mediate their effects via modulation of the Wnt/β-catenin pathway and more work is required to demonstrate mechanism, and the specific regulatory mechanisms between various signaling pathways also need to be further clarified in the future.

## Conclusions

In conclusion, our study showed that BMSC transplantation can relieve silica-induced pulmonary fibrosis in rats. We found that transplantation can reduced pathological changes to the lungs of silica-treated animals, restored epithelial characteristics, and decreased mesenchymal features as revealed by expression of specific proteins. Furthermore, attenuation of the Wnt/β-catenin signaling pathway may be one of the mechanisms involved in anti-fibrotic effects of BMSCs. The results provide new information on silica-induced pulmonary fibrosis and identify potential and possible targets for future therapy.

## Additional files

Additional file 1: The general morphology of fresh lung tissue in each group. (A) control group; (B) silica model group; (C) BMSC transplantation group; (D) BMSC-CM transplantation group. (TIF 1182 kb)

Additional file 2: Histological changes in lung tissue of rats on the 28th day after exposure to silica suspension in the control group and the silica model group, with magnification of 100 (left panel) and 200 (right panel). (A) H&E staining of lung tissue. Arrows indicate extensive aggregation of inflammatory cells and fibrotic lesions; (B) Masson staining of lung tissue. Arrows indicate the deposition of collagen fibers. (TIF 3622 kb)

## Abbreviations

BMSC-CM: Bone marrow mesenchymal stem/stromal cell-conditioned medium
BMSCs: Bone marrow mesenchymal stem/stromal cells
CT: Computed tomography
DMEM: Dulbecco’s modified Eagle’s medium
E-cad: E-cadherin
ECM: Extracellular matrix
EMT: Epithelial-mesenchymal transition
FCM: Flow cytometry
H&E: Hematoxylin and eosin
IPF: Idiopathic pulmonary fibrosis
SD: Sprague Dawley
SPF: Specific pathogen free
α-SMA: α-Smooth muscle actin
