# Supplementary figures and images for: Bone marrow mesenchymal stromal cells attenuate silica-induced pulmonary fibrosis potentially by attenuating Wnt/β-catenin signaling in rats
Source: Stem Cell Res Ther. 2018 Nov 14;9:311. doi: 10.1186/s13287-018-1045-4 (PMC6234553; doi:10.1186/s13287-018-1045-4)

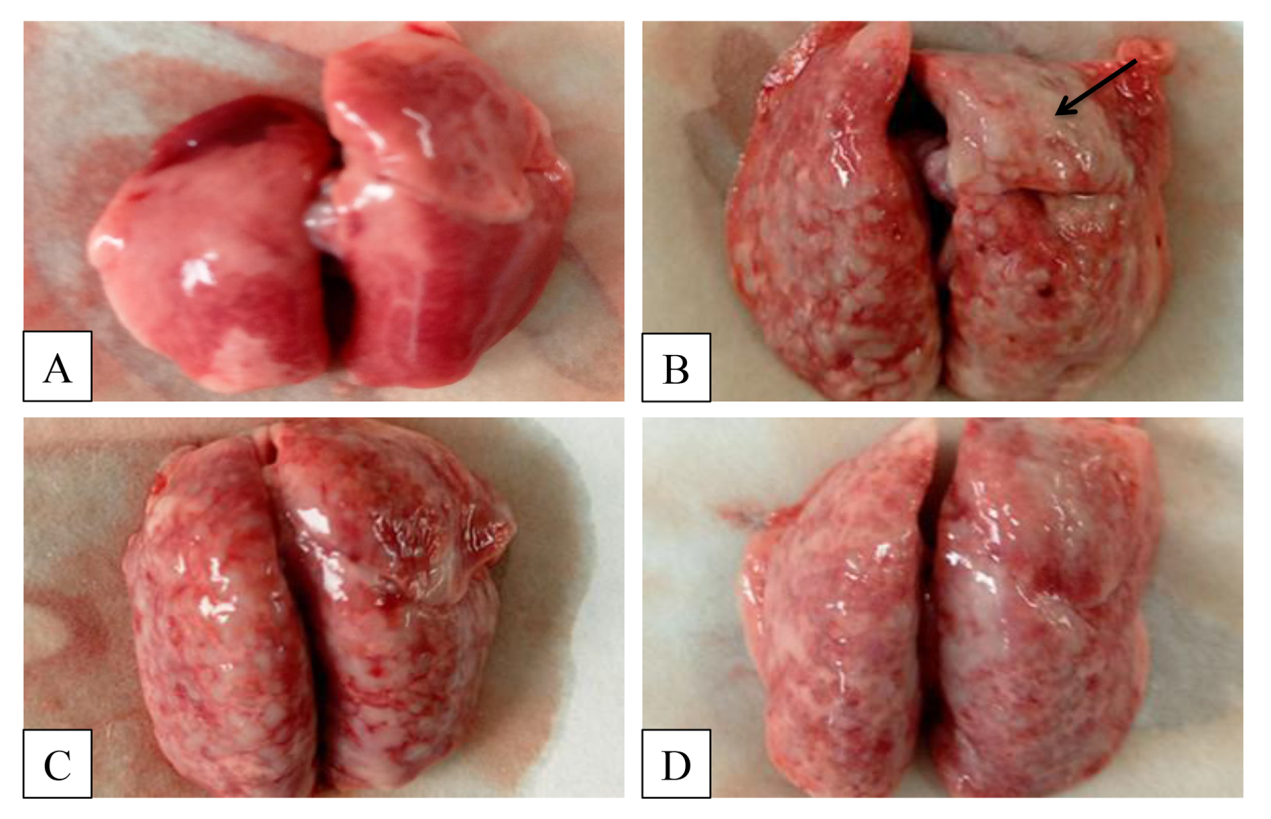

Supplement: Supplementary file 1 — The general morphology of fresh lung tissue in each group. (A) control group; (B) silica model group; (C) BMSC transplantation group; (D) BMSC-CM transplantation group. (TIF 1182 kb) [file 13287_2018_1045_MOESM1_ESM.tif]

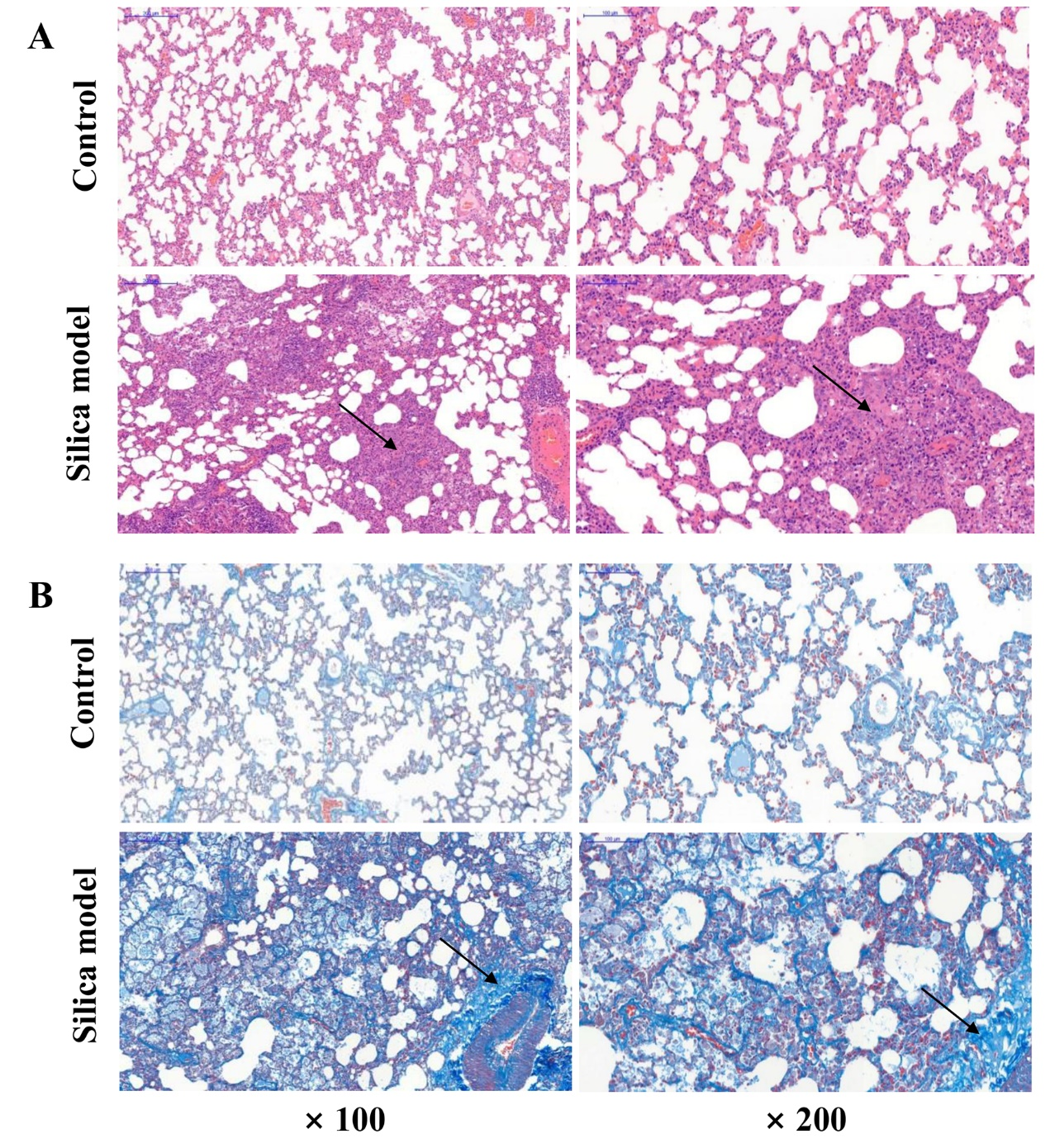

Supplement: Supplementary file 2 — Histological changes in lung tissue of rats on the 28th day after exposure to silica suspension in the control group and the silica model group, with magnification of 100 (left panel) and 200 (right panel). (A) H&E staining of lung tissue. Arrows indicate extensive aggregation of inflammatory cells and fibrotic lesions; (B) Masson staining of lung tissue. Arrows indicate the deposition of collagen fibers. (TIF 3622 kb) [file 13287_2018_1045_MOESM2_ESM.tif]
